# The Aging Waistline: Impact of the Geriatric Obesity Epidemic on an Urban Emergency Department: Original Communication

**DOI:** 10.4236/ijcm.2013.45047

**Published:** 2013-05

**Authors:** Heather M. Prendergast, Ernest Waintraub, Brad Bunney, Lisa Gehm, Carissa Tyo, Armando Marquez, John Williams, Angela Bailey, Diego Marquez, Marcia Edison, Mark Mackey

**Affiliations:** 1 Department of Emergency Medicine, University of Illinois, Chicago, USA; 2 Emergency Medicine Residency Program, University of Illinois, Chicago, USA; 3 Department of Medical Education, University of Illinois, Chicago, USA

**Keywords:** Elderly, Obesity, Emergency Department

## Abstract

**Purpose:**

Reviews adult emergency department (ED) visits for patients age 65 and older during one calendar year; determine the prevalence of weight classifications; identifies trends between BMI and discharge/admitting diagnoses, vital signs, and severity index.

**Methods:**

The electronic medical records system and data from the ED billing service was reviewed for an urban academic institution with an annual volume of 125,000 for patients age > 65. Using a random number table, a retrospective cohort of 328 elderly patients was selected for review, representing a convenience sample of 2.6% of elderly ED visits. Body Mass Index (BMI) was calculated, using the Center for Disease Control (CDC) formula with underweight (<18.5), normal (18.5 - 24.9), overweight (25 - 29.9), and obese (≥30).

**Results:**

The majority of the cohort in this study was African-American and Hispanic (60% and 27% respectively), and there were a higher percentage of females than males (60% and 40% respectively). Approximately 29% of the patients were classified as normal weight, 35% classified as overweight, and 36% as obese. The older the patient, the more likely that patient belonged to a lower weight classification (p < 0.01). Those presenting with neurological, pulmonary or gastrointestinal complaints were more likely to be of a higher weight classification (p < 0.05). Patients who were hypertensive on arrival to the ED were more likely to be in a higher weight classification (p < 0.01).

**Conclusion:**

Those patients with a higher weight classification had a strong correlation with selected abnormal vital signs and disease presentations. EDs are important sources of care for the elderly. EDs can serve as a previously untapped resource for screening and early referral exercise programs aimed at improving physical function/functional status and quality of life in the elderly patient population.

## 1. Introduction

### 1.1. Elderly and the ED Use

Patients 65 years of age or older represent an increasing proportion of emergency department (ED) visits [1]. Between 1993 and 2003, the number of elderly visiting the ED was higher than any other age group and this rate increased faster than any other age group [2]. Compared with younger persons, older adults use emergency services more, have longer ED visits, higher acuity, a higher percentage of admissions and/or repeat ED visits [3]. The impact of the elderly population on ED visits stems in part from the increase in their overall prevalence [4]. Baby boomers, those born between 1946 and 1964, number roughly 76 million children [4]. Those children born in 1946 will turn 65 and become elderly by 2011 [4]. By 2030 it is predicted there will be approximately 72 million elderly in our population [4].

### 1.2. Obesity among the Elderly

Obesity is a major public health concern. The increase prevalence of the elderly in the population has been accompanied by a concomitant increase in the prevalence of overweight and obesity in this age group [5]. For example, in the 1990's, when the prevalence of elderly was approximately 32 million, 26.1% of this population had a BMI over 30. By 2008, when the prevalence of elderly was approaching 40 million, 39.5% of this population had a BMI over 30 [6]. The economic implications of this are considerable. According to National Health Accounts Data, in 1998 approximately 23.5 billion dollars spent by Medicare were attributable to overweight and obesity, and 13.8 billion dollars on obesity alone [7,8]. It is projected that the more than 21% of the nation's direct health care spending in 2018 will be obesity-related direct expenditures, approximately $344 billion dollars [9].

Obesity has important functional implications in the older population, because it can exacerbate the age-related decline in physical function. Excess body fat mass and a BMI ≥ 30 in older subjects are associated with physical dysfunction and are predictive of a decline in functional status and future disability [10]. Moreover, older persons who are obese (BMI ≥ 30) have a greater rate of nursing home admissions than non-obese seniors (BMI: 18.5 - 24.9) [11].

A joint position statement from the American Society for Nutrition, the North American Association for the Study of Obesity (NAASO), and the Obesity Society highlights the need for a change from the traditional approaches to managing obesity. In the elderly, the primary goals of obesity treatment are centered around increasing physical functioning and quality of life, and not on disease prevention. Although emergency departments serve as a common entry point into the healthcare system for many elderly patients, the epidemic of geriatric obesity has not been well documented within the ED setting.

### 1.3. Statement of the Problem

The purpose of this research study was to examine emergency department visits for patients ages 65 or older to determine the prevalence of normal, overweight, and obesity in this population and to identifying associations between BMI and discharge/admitting diagnosis, vital signs, mode of arrival, severity index and disposition among the different groups. More specifically, answers to the following questions were sought:

- How does the prevalence of obesity in a random sampling of elderly ED population compare with national averages?
- Is there a difference between the chief complaint, vitals signs, ED disposition, and severity index when overweight and obese elderly are compared to normal-weight elderly?

## 2. Patients and Methods

### 2.1. Data Collection

A retrospective chart review from a single-center ED with an annual volume of 12,500 for patients age > 65 was conducted on a cohort of 328 elderly patients. The study period was one calendar year from January 1, 2009 to December 31, 2009. Elderly is defined as being age 65 or older. All elderly patients evaluated in the ED during the study time frame were eligible for inclusion in the study. The electronic medical records system and data from the ED billing service was reviewed. Using a random number table, a retrospective cohort of 328 elderly patients was selected for review, representing a convenience sample of 2.6% of the elderly ED visits. The study was reviewed and approved by the University of Illinois institutional review board. Charts were eliminated from final data analysis if any data pertinent to our study was missing.

Data collected from the electronic medical record included demographics, height/weight measurements, chief complaints, vital signs, mode of arrival, triage severity level and ED disposition. Height/weight measurements were either self-reported or obtained by the triage nurse. Body Mass Index (BMI) and percentiles were calculated using the Centers for Disease Control (CDC) formulas with normal BMI range (5th - 85th %ile), overweight or obese (≥85th %ile), and obese (≥95th %ile).

### 2.2. Setting

The emergency department is in a tertiary care academic institution in a largely African American and Hispanic neighborhood. The emergency department services both pediatric and adult patients with an annual adult volume of 46,000.

### 2.3. Statistical Analysis

Clinical characteristics and race/ethnicity were compared using Chi-square test/Fisher's exact tests for categorical variables. Logistic regression models were used to conduct pairwise comparisons. For all tests performed, percentages were reported and p-value < 0.05 was considered significant. SAS version 9.1 (SAS Institute Inc, Cary, North Carolina) was used for statistical analysis.

## 3. Results

The study cohort represented 328 unique elderly patient visits. Patients with multiple visits were only included in the database once. The majority of the patients were African American (197/60%) and Hispanic (87/27%), leaving (44/13%) in the Caucasian and other group (Table 1). More than half of each race was overweight or obese (African American 140/71%, Hispanic 66/76%, Caucasian/other 29/67% respectively) There were a higher proportion of females as compared to males (60% vs 40%), and 72% of females and 72% of males were of a higher weight classification. The most prevalent weight classification in the entire cohort was obesity at 36% followed by overweight at 35%. Normal weight made up only 29% of the cohort. As the patient group became older the prevalence of higher weight declined (p < 0.01, CI 0.918 - 0.984). Between the ages 65 and 74, the prevalence of overweight and obesity was 18% and 21% respectively. Between the ages 75 and 84 the prevalence of overweight and obesity was 13% and 11% respectively. The prevalence of overweight and obesity in those 85 years of age or older was 4% and 4%.

Patients with a neurological, pulmonary or gastrointestinal complaint were more likely to be in a higher weight classification (p < 0.05) (Table 2). Of the elderly with a neurological complaint (n = 52), 40% were overweight (n = 21) and 25% were obese (n = 13). Of the elderly with a pulmonary complaint (n = 45), 40% were overweight (n = 18) and 33% were obese (n = 15). Finally, of the elderly with a gastrointestinal complaint (n = 55), 40% were overweight (n = 22) and 25% were obese (n = 14).

Elderly patients who were hypertensive on arrival were more likely to be overweight or obese (p < 0.01) (Table 2). Of all those presenting to the emergency department who were hypertensive at triage, 76% were either overweight or obese. Specifically, 30% were overweight and 46% were obese.

There was no statistically significant association between admitted or discharged patients (Table 2) and the different weight classifications. Likewise, there was no statistically significant association between mode of arrival (Table 2), or severity index of care among obese, overweight, and normal weight elderly patients.

## 4. Discussion

The elderly visits to the ED are increasing in part because of their growing prevalence in the population. As the number of elderly increases there is a concomitant increase in the prevalence of overweight and obesity among them. The prevalence of overweight and obesity in our study is comparable to the prevalence in the general population [12]. This study found a positive correlation between overweight and obesity among the elderly in an urban ED and specific medical complaints. While many studies have shed light on this association in ambulatory clinics, this is the first study demonstrating an association in an ED setting [13-15].

The association observed in our study between increasing age among the elderly and declining prevalence of higher weight classification is consistent with data from large population studies showing that after age 60, mean body weight and BMI tend to decrease. This is in part explained by the survivor bias. Survivor bias is observed because overweight and obese individuals have a higher mortality risk at an earlier age [10]. Dr. Adams *et al*. demonstrated this phenomenon by showing that an increase in BMI among 50-year-old males and females who did not smoke increased the risk of death by 20 to 40 percent among overweight persons, and by two to three times among obese persons [16].

Our finding that hypertension was more likely among overweight and obese elderly patients has been corroborated in settings outside the ED also. Matsumura *et al.* studied 80-year-old patients and found that increases in BMI were associated with increases in mean systolic and diastolic pressure [17]. While the increases were significant for both sexes, an evaluation of whether sex influenced the relationship found the test for trend significant only for women [17].

## 5. Study Limitations

This study is a retrospective review conducted at a single academic ED and represented a convenience sample of 2.6% of eligible elderly visits. To confirm and generalize the results, a multicenter review is needed. Our study used BMI to determine whether higher weight classification is associated with increased risk of disease and abnormal vitals. The use of BMI for this purpose is not without critics [18]. BMI may overestimate body fat in the elderly who have kyphosis or compressed vertebral bodies. These conditions will decrease the height of a person and consequently increase BMI [10]. BMI may also underestimate body fat in the elderly by not taking into account body mass composition [10]. The CDC has acknowledged this and recommends taking other factors into account, such as waist circumference, when assessing disease risk [19]. We believe that while using waist circumference in our study may have shown a greater number of statistically significant correlations with disease, not using it did not weaken the associations we found using BMI. In addition, attempts to verify the accuracy of self-reported height/weight measurements was not routinely performed. Nonetheless, this study found significant correlations between elevated BMI and high-risk populations and confirms the magnitude of the obesity epidemic among the elderly.

## 6. Conclusion

The impact of the epidemic of obesity among the elderly has placed an unprecedented burden on geriatric health and will impact future healthcare costs, thus mandating the need for innovative strategies aimed at maximizing positive outcomes. Emergency departments continue to serve as important sources of care for the elderly in the United States. Moreover, EDs can serve as a previously untapped resource for screening and early-referral exercise programs aimed at improving physical functioning and quality of life.

## Figures and Tables

**Table 1:** Demographics of elderly study population. This table characterizes the study population by race/ethnicity.

Race	Number	Percent (%)
African-American	197	60
Caucasian/other	43	13
Hispanic	87	27

Other = Asian, Native American, American Indian.

**Table 2:** Chief complaints, disposition, mode of arrival and triage blood pressures by weight classification. This table compares chief complaint by organ system, disposition, mode of arrival, and triage vital signs for normal, overweight and obese patients.

Variable		(n) %	
**Ethnicity**	**Normal Weight (n = 92)**	**Overweight (n = 119)**	**Obese (n = 116)**
African-American	(57) 18	(66) 20	(74) 23
Caucasian/other	(14) 4	(17) 5	(12) 4
Hispanic	(21) 6	(36) 11	(30) 9
**Chief Complaint**	**Normal group (N = 117)**	**OW group (N = 157)**	**OB group (N = 136)**
Cardiovascular	(25) 8	(35) 11	(34) 10
General	(32) 10	(42) 13	(36) 11
Gastrointestinal*	(19) 6	(22) 7	(14) 4
HEENT	(3) 1	(6) 2	(7) 2
Infectious Dx	(1) 0.3	(3) 1	(1) 0.3
Musculoskeletal	(7) 2	(10) 3	(16) 5
Neurologic*	(18) 5	(21) 6	(13) 4
Respiratory*	(12)4	(18) 5	(15) 5
**Disposition (a)**	**Normal group (N = 91)**	**OW group (N = 117)**	**OB group (N = 111)**
Home/expired	(34) 10.5	(38) 12	(54) 17
General admit	(26) 8	(32) 10	(20) 6
Admitted to monitored bed	(31) 10	(47) 15	(37) 11.5
**Mode of arrival (b)**	**Normal group (N = 87)**	**OW group (N = 112)**	**OB group (N = 111)**
Ambulance	(28) 9	(30) 10	(32) 10
Car	(59) 19	(82) 26.5	(79) 25.5
Blood Pressure on arrival**	**Normal group (N =** 91**)**	**OW group (N =** 117**)**	**OB group (N =** 115**)**
Hypertensive	(46) 14	(59) 18	(90) 28
Hypotensive	(9) 3	(11) 3	(7) 2
Normotensive	(36) 11	(47) 15	(18) 6

* Testing the association between chief complaint and BMI classification (p-value = < 0.05) using Chi-square test;

** Patients with hypertension are more likely to be a higher weight classification than those not hypertensive (p-value < 0.01). (a) Testing the association between disposition and BMI classification (p-value = 0.838) using Chi-square test; (b) Testing the association between mode of arrival and BMI classification (p-value = 0.768) using Chi-square test.
